# Insuffisance rénale chronique et hémodialyse à Lomé: l´hémodialysé et son entourage sont-ils bien informés?

**DOI:** 10.11604/pamj.2021.39.85.26685

**Published:** 2021-05-28

**Authors:** Yawovi Mawufemo Tsevi, Badomta Dolaama, Komlan Georges Tona, Anani Amen Tevi, Edem Cruz Affanou, Amah Daniel Amede, Jannat Haroune-Traore, Yoan Eyram Makafui Amekoudi, Akomola Kossi Sabi, Weu Mélanie Tia, Delphine Amélie Lagou

**Affiliations:** 1 Service de Néphrologie et d´Hémodialyse du CHU Sylvanus Olympio de Lomé, Lomé, Togo,; 2 Service de Néphrologie du CHU de Kara, Kara, Togo,; 3 Service de Néphrologie du CHU de Bouaké, Bouaké, Côte d´Ivoire,; 4 Service de Néphrologie du CHU de Cocody, Cocody, Côte d´Ivoire

**Keywords:** Insuffisance rénale, chronique, terminal, hémodialyse, patients, connaissance, Lomé, Renal failure, chronic, end-stage, hemodialysis, patients, knowledge, Lomé

## Abstract

L´insuffisance rénale terminale nécessite un traitement de suppléance notamment l´hémodialyse. Avant l´initiation, le patient et son entourage reçoivent des informations sur la maladie rénale et les possibilités de traitement. Ce travail a pour objectif d´évaluer le niveau de connaissance ainsi que l´opinion de l´hémodialysé et de son entourage sur la maladie rénale chronique et les traitements de suppléances rénales. Il s´est agi d´une étude transversale descriptive qui s´est déroulée du 29 juillet au 19 août 2020 dans l´unité d´hémodialyse du CHU-SO. La population de notre étude était constituée de tous les patients hémodialysés de l´unité d´hémodialyse du CHU-SO et de leurs accompagnants qui ont donné leur consentement libre et éclairé. Les données ont été collectées de façon anonyme à partir d´un questionnaire. La saisie et l´analyse statistique des données ont été faites au moyen du logiciel Epi Info dans sa version 7.2.2.6. Quatre-vingt-et-un patients et 79 accompagnants ont été interrogés. Les âges moyens des patients et leur accompagnant étaient respectivement de 49,7 ans ± 13,5 et 39,6 ans ± 13,2. Tous les patients connaissaient leur pathologie et 94% des accompagnants, les informations sur la maladie de leurs parents. L´hémodialyse était considérée très chère par 95,1% des patients. L´avantage de l´hémodialyse le plus connu par les patients était la qualité de vie améliorée (80,2%) et 15% pensaient également que la transplantation rénale était tout aussi efficace. La majorité des accompagnants (85%) ont déclaré que l´hémodialyse était le meilleur traitement. L´éducation thérapeutique des patients hémodialysés et des accompagnants est importante pour une meilleure prise en charge globale des hémodialysés.

## Abstract (en)

End stage renal failure requires substitution therapy, including hemodialysis. Before initiation, patients and their entourage receive information on renal disease and treatment options. The purpose of this study is to assess the level of knowledge as well as the opinion of patients on haemodialysis and of their entourage on chronic kidney disease and substitution therapies. We conducted a cross-sectional descriptive study in the Department of Haemodialysis at the Sylvanus Olympio University Hospital (CHU-SO) from 29 July to 19 August 2020. The study population was composed by all the haemodialysis patients in the Department of Haemodialysis at the CHU-SO and by their accompanying persons who gave their free and informed consent. Anonymous survey was used to collect data. Data collection and statistical analysis were carried out using the Epi Info software 7.2.2.6. Eighty-one patients and 79 accompanying persons were interviewed. The average ages of patients and their accompanying persons were 49.7 years ± 13.5 and 39,6years ± 13.2 respectively. All patients knew their disease and 94% of accompanying persons were informed about their parents’ disease. Hemodialysis was considered very expensive by 95.1% of patients. Patients stated that improved quality of life was the most known benefit of hemodialysis (80.2%) while 15% also thought that renal transplantation was equally effective. The majority of accompanying persons (85%) stated that hemodialysis was the best treatment. Haemodialysis education program for patients and their accompanying persons is essential to better overall management of haemodialysis patients.

## Introduction

L´insuffisance rénale (IR) se définit comme une altération de la fonction émonctoire du rein [1]. Bien que très peu connue dans nos contrées, l´insuffisance rénale est une maladie avec une mortalité en nette croissance [2], une épidémie silencieuse progressant de façon alarmante, et concernant des millions d'êtres humains autour du globe. L´ampleur réelle de l´IR en Afrique subsaharienne reste inconnue par manque de registres nationaux. Au Togo, une fréquence hospitalière de 93,7% de patients présentant une insuffisance rénale chronique terminale (IRCT) avait été rapportée en 2019 en primo consultation de néphrologie [3]. L´hémodialyse (HD) constitue la base de la prise en charge de l´IRCT au Togo. Elle est presqu´entièrement à la charge du patient et de sa famille. Avant de débuter le traitement par hémodialyse, il est important d´informer et le patient et son entourage sur les avantages, les conditions requises, sur le déroulement des séances d´hémodialyse et sur les mesures hygiéno-diététiques. L´entourage, contribue en effet au ressenti du patient concernant l´hémodialyse. L´objectif général de ce travail est d´évaluer le niveau de connaissance ainsi que l´opinion de l´hémodialysé et de son entourage sur l´hémodialyse au Centre Hospitalier Sylvanus Olympio (CHU-SO). De façon spécifique, il s´est agi de: déterminer la connaissance ou non du diagnostic de leur maladie par les patients hémodialysés et leur accompagnant; évaluer la perception de l´hémodialyse concernant le cout, l´effet bénéfique sur leur état de santé; évaluer l´observance des traitements et le niveau de connaissance des traitements.

## Méthodes

Il s´est agi d´une étude transversale descriptive qui s´est déroulée pendant trois semaine allant du 29 Juillet au 19 Août 2020 dans l´unité d´hémodialyse du Centre Hospitalier Universitaire Sylvanus Olympio (CHU-SO) de Lomé, l´unique centre public d´hémodialyse au Togo. Cette unité a une capacité d´accueil de 81 patients. La population de notre étude était constituée des patients hémodialysés suivis dans l´unité d´hémodialyse du CHU-SO et de leurs accompagnants. Ont été inclus dans notre étude, tous les hémodialysés ainsi que les accompagnants qui ont donné leur consentement libre et éclairé. N´ont pas été inclus les hémodialysés et accompagnants ayant refusé la participation. Les données ont été collectées à partir de deux types de questionnaires préétablies: un adressé aux patients hémodialysés et l´autre à leurs accompagnants.

Les variables étudiées étaient: les données démographique: âge en année, sexe; le statut socio-économique : profession, revenu mensuel en franc CFA d´Afrique de l´Ouest; la source du financement des soins; les données de l´hémodialyse: le personnel médical ayant fait l´annonce du diagnostic de l´insuffisance rénale chronique, l´étiologie de l´IRCT selon le patient et le nombre de séance par semaine; la connaissance des avantages de l´hémodialyse et des autres traitements de suppléance rénale; l´opinion personnelle sur l´hémodialyse concernant le coût de la prise en charge, les effets bénéfiques ou non de l´hémodialyse. Définition opérationnelle; la profession a été classée en plusieurs groupes à savoir : fonctionnaire, élève et étudiants, commerçant, ménagère, ouvrier (personne effectuant un travail manuel), retraité, militaire et autres (toute personne ne répondant pas aux autres professions citées); le revenu a été évalué par rapport au Salaire Minimum Interprofessionnel Garanti (SMIG) qui est, selon la Convention Collective Interprofessionnelle du Togo entrée en vigueur le 06 Janvier 2012, fixant le salaire minimum (SMIG) à 35.000 F CFA, soit 53 Euros.

Nous avons alors classé en trois groupes: inférieur au SMIG, entre le SMIG et trois fois le SMIG (soit 100000 FCFA) et supérieur à trois fois le SMIG; l´observance a été évaluée sur le respect ou non des rendez-vous d´hémodialyse; l´opinion personnelle a été évaluée en se basant sur les avantages ressenties par le patient depuis l´initiation de l´hémodialyse; un accompagnant a été défini comme une personne ayant un lien familial ou non avec un patient hémodialysé et l´accompagnant au centre d´hémodialyse.

Les patients suivis en hémodialyse étaient reçus individuellement en entretien de même que leurs accompagnants pour la collecte des informations. La saisie et l´analyse statistique des données ont été faites au moyen du logiciel Epi Info dans sa version 7.2.2.6 afin de déterminer la prévalence des variables d´étude. Les données ont été collectés dans l´anonymat avec le consentement libre et éclairé de nos enquêtés. Afin de minimiser les biais, le questionnaire a été remis au patient seul durant la séance de dialyse, puis à son accompagnant dans la salle d´attente de l´hémodialyse. Nous n´avons pas pu obtenir l´accord du comité de Bioéthique avant le début de la collecte des données.

## Résultats

**Résultats patients:** tous les patients hémodialysés soit 81 (100%) ont été inclus.

**Caractéristique sociodémographique:** l´âge moyen était de 49,7 ans ± 13,5 avec des extrêmes de 18 et 80 ans. Le sex ratio homme-femme était de 1,5 (Tableau 1).

**Tableau 1:** description des caractéristiques concernant les patients hémodialysés à Lomé en 2020

Caractéristiques	Effectif (N=81)	Pourcentage (%)
Niveau d´étude		
Primaire	9	11,1
Secondaire	38	46,9
Universitaire	27	33,3
Non scolarisé	7	8,6
Revenu		
< SMIG	20	25
35000-100000	42	52,5
>100000	18	22,5
Causes d´IRCT selon le patient		
Diabète	36	44,4
Hypertension artérielle	58	71,6
Drépanocytose	4	4,9
Paludisme	9	11,1
Anémie	3	3,7
VIH	9	11,1
Polykystose rénale	5	6,2
Autres	8	9,9
Nombre de séances par semaine		
Une	2	2,5
Deux	77	95
Trois	2	2,5
Profession		
Fonctionnaire	14	17,5
Élève et étudiants	4	5
Commerçant	17	21,2
Ménagère	12	15
Ouvrier	2	2,5
Retraité	13	16,2
Militaire	2	2,5
Autres	16	20
Financement des soins		
Famille	63	77,8
Ami	6	7,4
Assurance	8	9,9
ONG	4	4,9

SMIG: Salaire Minimum Interprofessionnel Garanti; IRCT: Insuffisance Rénale Chronique Terminale; VIH: Virus de l´Immunodéficience Humaine; ONG: Organisation Non Gouvernementale

### Connaissances des patients sur leurs états cliniques

Tous les malades affirmaient savoir de quoi ils souffraient. Le diagnostic de l´insuffisance rénale chronique terminale a été fait dans presque tous les cas par un médecin et 93,4% avaient reçu des informations et conseils auprès du médecin traitant au moment du diagnostic. Les caractéristiques des patients concernant le niveau d´étude, le revenu, les causes d´IRCT selon le patient, le nombre de séances par semaine, la profession et le financement des soins sont détaillées dans le Tableau 1. La plupart des patients (85,2%) payait 34000 Francs CFA (52€) par séance et 65,4% dépensaient en moyenne entre 300.000 et 1.000.000 de Francs CFA par mois y compris les médicaments (antihypertenseurs oraux, la supplémentation en Fer, de l´érythropoïétine, les antibiotiques), le matériel d´abords vasculaires et leur transport. Ceux qui dépensaient moins de 300.000 Francs CFA représentaient 29,6% et 5% dépensait plus de 1.000.000 Francs CFA. Pratiquement tous les patients (95,1%) estimaient que la dialyse revenait très chère.

### Observance du traitement et avantage de l´hémodialyse

La plupart d´entre eux (85,7%) respectait les rendez-vous d´hémodialyse contre 14,3% qui ne le respectaient pas pour manque de moyen financier. Soixante-neuf pourcent des patients étaient soulagés après leur séance de dialyse. Les avantages de l´hémodialyse les plus connus par les patients étaient : la qualité de vie améliorée (80,2%), un meilleur confort (22,2%). Cependant 15% pensaient également que la transplantation rénale était tout aussi efficace.

### Résultats accompagnants

#### Caractéristique sociodémographique

L´âge moyen était de 39,6 ans ± 13,2 avec des extrêmes de 16 et 69 ans. Le sex ratio homme-femme était de 1,1. Les caractéristiques concernant le niveau d´étude, la profession, le revenu des accompagnants sont résumées dans le Tableau 2.

**Tableau 2:** caractéristiques des accompagnants des hémodialysés à Lomé en 2020

	Effectif (N=79)	Pourcentage (%)
Niveau d´étude		
Primaire	6	7,6
Secondaire	42	53,2
Universitaire	26	33
Non scolarisé	5	6,2
Profession		
Fonctionnaire	10	12,6
Élève et étudiants	14	17,7
Commerçant	16	20,2
Ménagère	18	22,8
Ouvrier	5	6,3
Retraité	3	3,9
Militaire	3	3,9
Autres	10	12,6
Revenu		
<SMIG	29	36,7
35000-100000	35	44,3
>100000	15	19

SMIG: Salaire Minimum Interprofessionnel Garanti

Connaissances des accompagnants sur l´état clinique des hémodialysés et les différents facteurs de risque de l´insuffisance rénale: concernant leur connaissance sur la maladie de leurs patients, 94,8% des accompagnants avaient répondu “Oui”.

### Traitement de l´insuffisance rénale chronique terminale

La majorité des accompagnants (85%) ont déclaré que l´hémodialyse était le meilleur traitement. Seulement 19,2% des accompagnants ne connaissait pas les avantages de l´hémodialyse. Les 80,8% restant avaient cités respectivement comme avantage: la qualité de vie améliorée dans 59% des cas et le confort du malade dans 31% des cas.

## Discussion

A notre connaissance, il s´agit de la toute première étude portant sur l´évaluation du niveau de connaissance des patients hémodialysés et de leurs entourages sur l´insuffisance rénale chronique, ses facteurs de risques ainsi que le traitement de suppléance. Notre étude a montré que la population des hémodialysés et des accompagnants était jeune au Togo. L´âge moyen de nos patients (49,7 ans ± 13,5) et celui des accompagnants était de (39,6 ans ± 13,2). En effet cet âge est similaire à ceux retrouvés en Afrique noire par Sabi *et al*. [4], Tsevi *et al*. [5], Ndiaye [6] et, Lengani *et al*. [7] qui ont respectivement rapporté des âges moyens de 49,5 ans, 38,8 ans, 44,73 ans, 40 ans pour les patients. Au Maghreb, Noto-kadou *et al*. [8] ont rapporté un âge moyen de 41,4 ± 12 ans chez les hémodialysés et 44 ± 10,5 chez les accompagnants. Cependant, des travaux réalisés dans les pays occidentaux ont montré que les patients en hémodialyse chronique étaient plus âgés, au-delà de 60 ans [9, 10]. Cette différence peut être liée à l´avancée médicale des pays développés, entrainant une espérance de vie plus longue avec des pyramides des âges plus larges au sommet. Elle pourrait également être due à la non maitrise des facteurs de risque cardiovasculaires, pourvoyeurs d´insuffisance rénale terminale dans les pays en développement. La majorité des patients (83,8%) étaient des travailleurs. Ndiaye *et al*. [6] au Sénégal avaient retrouvé 68,7% de patients inactifs. Cette différence pourrait résulter d´un biais dans notre sélection des patients. En effet, les séances de dialyse étant onéreuses (34 000 FCFA / séance de dialyse soit 52,44 €), très proche du salaire minimum interprofessionnel garanti (SMIG = 35 000 FCFA soit 54 €), seuls les patients travailleurs arrivaient à être observant. Elle pourrait aussi s´expliquer par l´inégalité de taille de l´échantillon.

La majorité des patients hémodialysés (94%) et leurs accompagnants (94,8%) avaient déclaré avoir reçu d´information concernant l´hémodialyse. En effet ceci est dû au fait que notre unité d´hémodialyse appartient à un service spécialisé en néphrologie donc nos patients devraient d´avantage être informé sur l´hémodialyse. Cependant, on constate que nos patients ont moins d´informations sur la transplantation rénale parce que dans la sous-région et chez nous au Togo, la transplantation rénale est rare. Ces résultats sont superposables au travail de Noto-Kadou *et al*. [8] qui rapportait une méconnaissance sur la transplantation rénale estimé à 62,7% chez les patients hémodialysés et 61,4% chez les accompagnants. Laouad *et al*. [11] dans une étude similaire mais multicentrique menée dans trois centres d´hémodialyse du Maroc en 2011 avait rapporté un taux de méconnaissance de 52,5%. Tous les patients et accompagnants ont déclaré que l´hémodialyse coutait très cher (34000 FCFA par séance de dialyse soit 52,44 €). En Côte d´Ivoire, le coût de la séance de l´hémodialyse est de 1750 FCFA. Cette différence de prix peut s´expliquer par l´absence d´assurance maladie universelle, le niveau socio-économique bas et la faible subvention de l´État au Togo. Il faut en outre ajouter que toutes les autres dépenses liées aux traitements tels que les médicaments antihypertenseurs, l´héparine, l´érythropoïétine et le fer injectable sont également à la charge des patients.

### Étiologies et facteurs de risque de l´insuffisance rénale chronique

Les causes de l´IRC cités par les patients étaient: l´HTA dans 61,6% des cas, diabète dans 44% des cas, polykystose rénale dans 6,2% des cas, médicaments traditionnels dans 44,3% des cas, VIH dans 11,1% des cas et le tabac dans 30,3% des cas. En effet Benja *et al*. [12] avaient rapporté que 66,95% de patients atteint d´insuffisance rénale avaient deux facteurs de risque cardiovasculaire : HTA était rencontré dans 59,83% des cas et le tabagisme dans 38,49% des cas. Selon la littérature, plus il existait de facteurs de risques cardiovasculaire, plus la fréquence de l´insuffisance rénale est élevée [13-15]. Tsevi *et al*. [5] dans une étude similaire ont rapporté des facteurs de risques de l´insuffisance rénale chronique tels que : HTA dans 66,1% des cas, diabète dans 10,2% des cas, VIH dans 20,3% des cas, polykystose rénale dans 1,7% des cas, tabagisme dans 5,1% des cas, médicament traditionnel dans 62,5% des cas. La forte prévalence de ces facteurs de risques dans nos pays pourrait s´expliquer par la toxicité des médicaments traditionnel, l´automédication et une mauvaise condition de vie alimentaire telle que la consommation excessive de sel, de sucre, d´huile; du tabac et de l´alcool, qui sont responsable des maladies cardiovasculaires (HTA, Diabète), précurseur des maladies rénales chroniques.

## Conclusion

Au Togo, l´hémodialyse est le traitement de base des patients souffrant de l´insuffisance rénale chronique terminale. L´éducation thérapeutique des patients hémodialysés et des accompagnants est importante pour une meilleure prise en charge globale des hémodialysés. Cependant, ce traitement reste encore très onéreux.

### Etat des connaissances sur le sujet

- Les patients hémodialysés sont peu satisfaits de la qualité des soins reçus;
- Aucune étude n´a été réalisée pour évaluer le niveau de connaissance de l´insuffisance rénale chronique et de son traitement.

### Contribution de notre étude à la connaissance

- Les hémodialysés à Lomé sont en majorité informés de leur maladie et du traitement par l´hémodialyse;
- Peu de patients et leur entourage sont informés des avantages et inconvénients de la transplantation rénale;
- La principale difficulté rencontrée par les patients est le cout élevé de la prise en charge.
